# Long-Term Impact of Economic Downturn and Loneliness on Psychological Distress: Triple Crises of COVID-19 Pandemic

**DOI:** 10.3390/jcm10194596

**Published:** 2021-10-06

**Authors:** Shuyan Liu, Matthias N. Haucke, Stephan Heinzel, Andreas Heinz

**Affiliations:** 1 Department of Psychiatry and Psychotherapy, Campus Charité Mitte, Charité—Universitätsmedizin Berlin, 10117 Berlin, Germany; andreas.heinz@charite.de; 2 Clinical Psychology and Psychotherapy, Department of Education and Psychology, Freie Universität Berlin, 14195 Berlin, Germany; stephan.heinzel@fu-berlin.de

**Keywords:** mental health and wellbeing, income, government debt, perceived social isolation

## Abstract

Background: The COVID-19 crisis poses global mental health and global economy challenges. However, there is a lack of longitudinal research investigating whether financial instability and social disruption may increase the risk of developing mental health problems over time that may potentially outlast the pandemic. Methods: We conducted an online survey for members of the general population (*n* = 2703) in Germany during the twelve months spanning from April 2020 to March 2021. We investigated the development of COVID-19 related psychological distress, the number of unemployed people, federal government debt, income distribution, and loneliness over the time period. Results: Over a period of twelve months, 53.6% of respondents in Germany reported experiencing psychological distress, varying from mild levels, 34.2%, to severe levels, 19.4%, of distress. High federal government debt, high incident COVID-19 cases, low incomes, and the prevalence of loneliness were found to be associated with increased long-term mental health problems. Psychological distress scores were most strongly increased in female and young respondents as well as those who reported fewer years of education, low income, and higher loneliness. Conclusions: Our study highlights factors that have a long-term impact on mental health amid the COVID-19 pandemic. We suggest that specific mental-health services could be offered to support high-risk groups experiencing financial fragility and loneliness. For purposes of safeguarding their mental health there is a need to monitor and track such risk factors in real time.

## 1. Introduction

The COVID-19 pandemic poses a great challenge for global mental health [1,2,3] and calls for systemic responses to address mental health needs [4]. Public health strategies such as quarantine, social distancing, and isolation, limit the transmission of COVID-19. However, the economic and social costs of these strategies are substantial, as unemployment has surged in many countries, economic and trade activities have been severely affected, and social risks and vulnerabilities have emerged [5,6]. Moreover, social isolation brings on or exacerbates mental health problems [7,8,9]. Individuals feel fear, sadness, anxiety, and face worry and isolation [4]. A meta-analysis of 50 studies amid COVID-19 showed that the pooled prevalence of psychological problems included poor sleep quality (40%), stress (34%), psychological distress (34%), insomnia (30%), post-traumatic stress symptoms (27%), anxiety (26%) and depression (26%) [10]. The burden of these psychological problems was highest among COVID-19 patients followed by healthcare workers and the general population [10]. However, another recent meta-analysis revealed a higher prevalence of suicidal thoughts/ideation or self-harm (11% vs. 5.8%) among the general public compared to healthcare professionals [11]. In addition, COVID-19 exacerbates pre-existing mental health problems as well as neurological disorders and substance use disorders through the limited access to healthcare services [4,12]. Therefore, the potential association between the long-term economic and mental health consequences of COVID-19 requires further elucidation.

Previous research reported that risk factors associated with mental health problems amid COVID-19 include being female, being of a younger age, student status, unemployment, and the presence of sleep disruptions/mental disorders [13,14]. In addition to these risk factors, we found that loneliness and unemployment had a negative impact on mental health during the first 6 months of the pandemic [15].

During the pandemic, COVID-19 associated high unemployment levels and rising debt affected the global economy and led to fears of an impending economic crisis and recession [5,16]. An economic recession can exacerbate mental health problems through growing socioeconomic risk factors such as unemployment, low income and financial strain, and debt [17]. There has been relatively little research into debt as a specific socioeconomic risk factor for poor mental health compared to unemployment, income, and financial strain [18,19,20]. On the one hand, rising government debt is a good way to obtain extra funds to invest in economic growth and productive employment in the short run [21]. On the other hand, unsustainable government debt accrued during the COVID-19 pandemic may raise interest rates and lower national income, which can lead to a fiscal crisis in the medium/long run [22]. In addition to debt, one study explored the initial impact of the pandemic on economic wellbeing, and found that about 20% of respondents were financially fragile [23]. Younger respondents and those with low income were particularly disadvantaged, having far less capacity to deal with their health and financial fragility [23]. Social causation hypothesis suggests that experiencing economic hardship increases the risk of subsequent mental health problems [24]. Economic adversities during a global economic downturn have indeed been associated with a long-lasting decline in mental health [25]. Another study also supported the social causation hypothesis and found that economic hardship experienced during the early months of the pandemic was detrimentally associated with psychological distress [26].

Moreover, income is a consistent predictor of social isolation and loneliness, and people with a lower income tend to experience greater isolation and less of a sense of belonging than people with a higher income [27]. During COVID-19, low-income individuals are particularly at risk of experiencing high levels of loneliness and subsequently, poor mental health [28]. A recent study found that younger females and individuals with lower income experience greater loneliness and mental health challenges [29].

So far, it is still unclear how general socioeconomical stressors (e.g., federal government debt, the number of unemployed people, and COVID-19 active cases) and individual socioeconomical stressors (individual income and loneliness level) contribute to psychological distress. Longitudinal data may be well suited to identify those risk factors associated with developing mental health problems and for determining intervention targets. Our study provides important evidence, which has not been reported previously, regarding the long-term (twelve-month) impact of general and individual socioeconomical stressors on psychological distress.

We aimed to examine whether self-reported psychological distress increased during the pandemic simultaneously with increasing general and individual socioeconomical stressors. In line with the social causation hypothesis, we assumed that federal government debt, the number of unemployed people, and COVID-19 active cases in the country predicts self-reported psychological distress over a twelve-month period. Furthermore, we hypothesized that individual low-income and high levels of loneliness predict the development of psychological distress. Lastly, we expected there to be differences in gender, age, and educational level in the prevalence of psychological distress.

## 2. Materials and Methods

### 2.1. Participants and Procedure

We created an anonymous online survey using the Siuvo platform (https://www.siuvo.com, accessed on 27 September 2021). The Siuvo platform is an expert-level artificial intelligence platform for psychological assessments in healthcare settings. We distributed our survey using a QR-code and shared it primarily via social media, advertisements, and newsletters. Data were collected in Germany from April 2020 to March 2021. The number of active cases of COVID-19 (i.e., by removing deaths and recoveries from total cases) can be found on the COVID-19 information page on the Worldometer website (https://www.worldometers.info/coronavirus/country/germany/, accessed on 27 September 2021).

Our online survey consisted of a socio-demographic assessment (i.e., gender, age, years of education), the COVID-19 Peritraumatic Distress Index (CPDI) questionnaires, and the short-form UCLA Loneliness Scale (ULS-8) questionnaires. The study was approved by both the Ethics Committee of Charité—Universitätsmedizin Berlin (ref: EA2/143/20) and the Ethics Committee of Freie Universität Berlin (ref: 030/2020).

### 2.2. Psychological Distress and Loneliness

We used the COVID-19 Peritraumatic Distress Index (CPDI) to obtain the level of psychological distress among general populations [30,31]. The CPDI has been validated across multiple countries [13,32]. There are 24 items and each item is rated on a 5-point scale ranging from 0 (“strongly disagree “) to 4 (“strongly agree”). A score below 28 indicates no distress, scores between 28 and 51 indicate mild to moderate distress, and scores above 52 indicate severe distress. Moreover, we used a short 8-item UCLA Loneliness Scale (ULS-8) to measure an individual’s subjective perception of loneliness or social isolation [33]. The detailed items of the CPDI and ULS-8 questionnaires can be found in our previous study [15].

### 2.3. Annual Income, Monthly Federal Government Debt, and Unemployment

Respondents reported their annual net income based on 12 income categories (€0–€4.999, €5.000–€9.999, €10.000–€14.999, €15.000–€24.999, €25.000–€49.999, €50.000–€74.999, €75.000–€99.999, €100.000–€124.999, €125.000–€149.999, €150.000–€174.999, €175.000–€200.000, and >€200.000). For a group comparison of income levels (higher vs. lower), each respondent’s income was ranked as higher versus lower than the median.

We obtained data on the average monthly federal government debt throughout Germany from the Federal Republic of Germany—Finance Agency (https://www.deutsche-finanzagentur.de/en/finance-agency/publications/, accessed on 7 June 2021). We acquired data on the number of unemployed people in Germany from the Federal Employment Agency of Germany (https://de.statista.com/statistik/daten/studie/1319/umfrage/aktuelle-arbeitslosenzahl-in-deutschland-monatsdurchschnittswerte/, accessed on 1 July 2021).

### 2.4. Data Analysis

We tested the following hypotheses: (1) variables indicating an economic downturn (federal government debt and the number of unemployed people as proximal predictors) and the number of active COVID-19 cases in the country are associated with self-reported psychological distress; (2) individual low income and high loneliness levels are associated with self-reported psychological distress; and (3) people who are female, young, have fewer years of education, low income, and report high loneliness are particularly at risk of experiencing psychological distress amid the pandemic.

Statistical tests were performed in the R version 4.1.0. system (www.r-project.org, accessed on 10 August 2021). Differences were considered as statistically significant at *p* < 0.05 and highly statistically significant at *p* < 0.001. We used a one-way ANOVA to explore sociodemographic differences (i.e., gender, age, and education) between the twelve-month groups, with respect to each month in order to control any potential confounding effects. Through ANOVA, we used “month” (each month from April 2020 to March 2021) as the independent variable, and “gender”, “age”, and “education” as the dependent variables. In accordance with our hypotheses, we tested whether general stressors (e.g., federal government debt, unemployment, and COVID-19 active cases) were associated with an increase in psychological distress over time. We built up a multiple linear regression model (“federal government debt”, “the number of unemployed people”, “the number of active COVID-19 cases”, and “month” as predictors and “CPDI distress scores” as the outcome) and controlled for sociodemographic differences (i.e., gender, age, and education). To meet the assumption of having no multicollinearity in a multiple regression, we calculated the variance inflation factor (VIF) values for all independent variables of the model. Secondly, to assess whether individual socioeconomical stressors (individual income and loneliness levels) had an influence on psychological distress, we performed a multiple linear regression analysis. We used “CPDI distress scores” as the outcome, and “ULS-8 loneliness scores”, “income levels” as well as their interactions between “ULS-8 loneliness scores” and “income levels” as predictors, controlling for “gender”, “age” and “years of education” for the analyses. Thirdly, to determine differences between groups above versus below the respective mean value of interest (age, education, income, and loneliness levels), we divided participants into two groups using the median split. We then calculated the difference between groups using an independent *t*-test for effects of gender (males versus females), age (elder versus younger), education levels (higher vs. lower), income levels (higher vs. lower), and loneliness levels (higher vs. lower), for which two-tailed *p* values were assumed.

## 3. Results

### 3.1. Group Description

There were 2703 respondents in Germany (2004 females; age range: 18–81, Mean = 36.45, SD = 13.28) who participated in our survey from April 2020 to April 2021. Over these twelve months, in Germany, there have been an average of 122,262 active COVID-19 cases per day, which reached its peak with an average of 356,223 active cases in December 2020 (with a peak number of 400,245 cases on 24 December 2020). Every participant took the survey once. Participants’ sociodemographic variables and group comparisons are presented in Table 1. Regarding sociodemographic variables between the groups assessed during each of the 12 months, we did not find a significant difference in “gender” (*F* (11, 2691) = 1.50, *p* = 0.123), but we did find significant differences in “education” (*F* (11, 2589) = 20.12, *p* < 0.001) and “age” (*F* (11, 2683) = 34.97, *p* < 0.001) among the twelve groups. In general, CPDI scores differed in relation to gender, age, and education levels. Female respondents experienced relatively higher levels of psychological distress compared to males, *t* (2701) = −3.53, *p* < 0.001. Younger respondents experienced relatively higher levels of psychological distress compared to older respondents, *t* (2630) = −11.24, *p* < 0.001. Respondents with fewer years of education, experienced relatively higher levels of psychological distress compared to respondents with more years of education, *t* (2269.611) = −10.50, *p* < 0.001, as shown in Table 1.

### 3.2. Increased Psychological Distress Associated with High Federal Government Debt over the Course of the Pandemic in Germany

**Figure 1:**
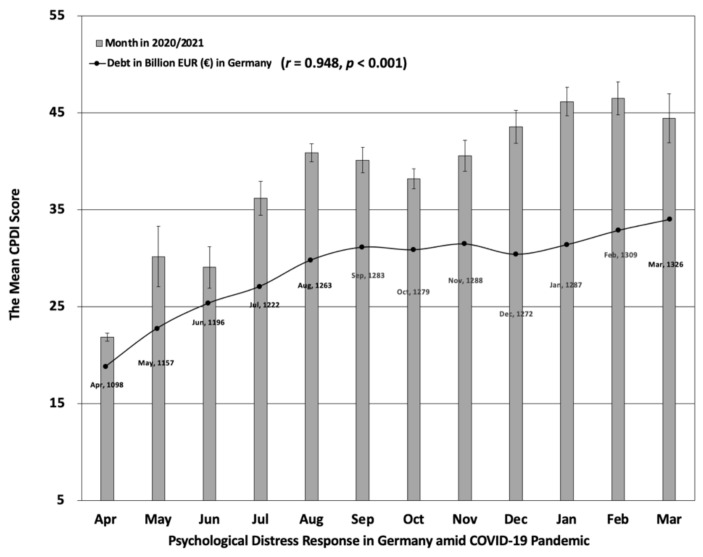
Psychological distress response and federal government debt (as an indicator of economic challenges) in Germany between April 2020 and March 2021. Error bars represent standard errors of the mean. Data of federal government debt in Germany retrieved from the Federal Republic of Germany–Finance Agency (https://www.deutsche-finanzagentur.de/en/finance-agency/publications/, accessed on 7 June 2021).

We found that self-reported psychological distress increased over the course of the pandemic in Germany (Pearson *r* = 0.911, *p* < 0.001, two-tailed), as shown in Figure 1. From April 2020 to March 2021, 53.6% of respondents in Germany reported psychological distress, varying from mild distress, which was at 34.2%, to severe distress which was at 19.4%, with an average COVID-19 Peritraumatic Distress Index (CPDI) score of (Mean) 33.59 (SE = 19.16). The average levels of psychological distress associated with the COVID-19 pandemic significantly rose from 26.0% (varying from 22.1% mild to 3.9% severe) in the first three months (between April and June 2020) up to 81.0% (varying from 41.0% mild to 40.0% severe) in the last three months (between January and March 2021) of our survey.

In a multiple regression model of general socioeconomic factors, we found that “federal government debt” (*β* = 0.30, *t* (2591) = 8.65, *p* < 0.001), “the number of unemployed people” (*β* = 0.14, *t* (2591) = 4.26, *p* < 0.001) and “the number of active COVID-19 cases” (*β* = 0.09, *t* (2591) = 4.15, *p* < 0.001) statistically significantly predicted psychological distress by controlling for sociodemographic differences (i.e., gender, age, and education). There were no significant associations between the three variables “federal government debt”, “the number of unemployed people”, and “the number of active COVID-19 cases” (all *p* values > 0.107, VIF values < 4.23). An increase in federal government debt (Pearson *r* = 0.948, *p* < 0.001, two-tailed) and the number of active COVID-19 cases in Germany (Pearson *r* = 0.599, *p* = 0.040, two-tailed) were found to be statistically significantly associated with increased perceived distress, while the number of unemployed people in Germany was not significantly associated with increased perceived distress (Pearson *r* = 0.427, *p* = 0.167, two-tailed). Due to a higher proportion of female participants, we also conducted additional analyses of males and females separately. We found that psychological distress was significantly predicted by “federal government debt” as well as “the number of unemployed people” in both males and females. In male participants, the predictors “federal government debt” (*t* (667) = 4.79, *p* < 0.0001) and “the number of unemployed people” (*t* (667) = 1.98, *p* = 0.048) statistically significantly increased psychological distress. Also in female participants, the predictors “federal government debt” (*t* (1919) = 7.17, *p* < 0.0001) and “the number of unemployed people” (*t* (1919) = 3.84, *p* < 0.0001) statistically significantly increased psychological distress.

### 3.3. Low-Income and Lonely Respondents Are at High Risk to Experience Psychological Distress

**Figure 2:**
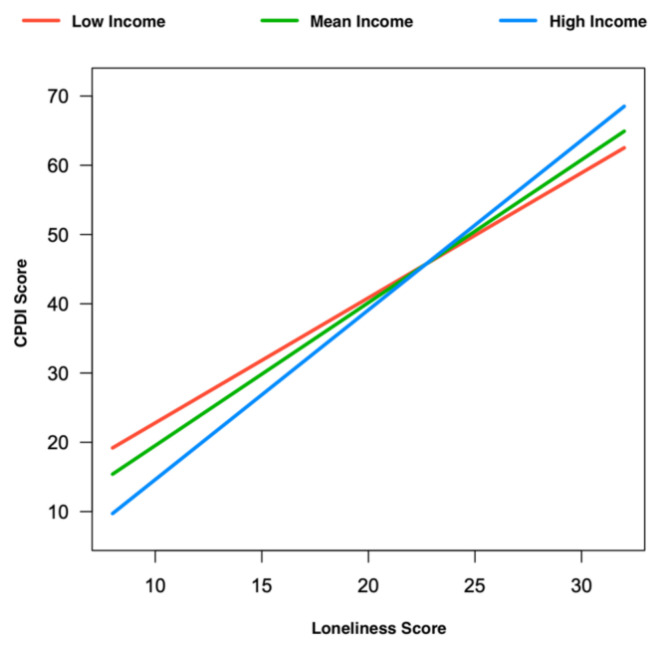
The effect of loneliness and income on psychological distress response in Germany during COVID-19 pandemic. The CPDI score, capturing psychological distress among general populations, is displayed as a function of loneliness (ULS-8 score) for high income in blue versus low income in red. Each participant’s self-reported annual net income was ranked higher versus lower than the median (*n* = 1193/1068: 442 subjects’ data in the median (category of €15.000–€24.999) were removed).

Regarding individual differences, we found the significant main effects of income (*b* = −2.30, *t* (1087) = −3.36, *p* < 0.001) and loneliness (*b* = 1.70, *t* (1087) = 13.44, *p* < 0.001) and their interaction (*b* = 0.11, *t* (1087) = 3.60, *p* < 0.001) on psychological distress by controlling for sociodemographic differences (i.e., gender, age, and education). Low income was significantly associated with high loneliness (Pearson *r* = −0.060, *p* = 0.014, two-tailed). As shown in Figure 2, individuals with lower incomes experienced higher psychological distress (mean = 38.71, SE = 18.07) compared to those with higher incomes (mean = 28.74, SE = 18.78). Individuals who experience higher levels of loneliness also reported higher psychological distress (mean = 52.43, SE = 16.00) compared to those who experienced lower levels of loneliness (mean = 29.84, SE = 15.33). Due to a high proportion of female participants, we also conducted additional analyses of males and females separately. We found the same results in both males and females. In male participants, we found the significant main effects of income (*b* = −4.14, *t* (275) = −2.94, *p* = 0.004) and loneliness (*b* = 1.50, *t* (275) = 5.94, *p* < 0.001) and their interaction (*b* = 0.18, *t* (275) = 2.71, *p* = 0.007) on psychological distress. In female participants, we also found significant main effects of income (*b* = −1.71, *t* (807) = −2.18, *p* = 0.03) and loneliness (*b* = 1.78, *t* (807) = 12.02, *p* < 0.001) and their interaction (*b* = 0.09, *t* (807) = 2.51, *p* = 0.01) on psychological distress.

## 4. Discussion

We conducted a 12-month study to evaluate the impact of general and individual economic challenges as well as loneliness on the prevalence of psychological distress. Specifically, we examined federal government debt, the number of unemployed people, COVID-19 active cases, individual income and loneliness levels and their respective impacts on psychological distress. In line with our hypothesis, we found that increased federal government debt, a high incident of COVID-19 cases, low income, and high levels of self-reported loneliness were associated with increased psychological distress over 12 months of the pandemic in Germany. Females and young people as well as those with fewer years of education, lower income, and people experiencing higher levels of loneliness were particularly at risk for experiencing psychological distress. The pandemic may therefore be particularly challenging for younger people who are still in education and training which means that they have fewer years of education and have a lower income because they are pursuing their education instead of already working a full-time job.

Our findings support the social causation hypothesis [24] by showing that both lower individual income as well as a generally increased federal government debt contributed to increased psychological distress over 12 months of the pandemic in Germany. This is also consistent with some previous studies suggesting that high government debt can hamper growth, and that countries with high government debt are vulnerable to adverse shocks [16,22,34]. On the other hand, an increase in government debt can stimulate economic growth via the resultant employment generation and through productive investment [21]. However, this relationship is only applicable in the short-term [21]. Federal government debt in Germany increased over time from €1098 billion euro in April 2020 to €1326 billion euros in March 2021 (https://www.deutsche-finanzagentur.de/en/finance-agency/publications/, accessed on 7 June 2021), reflecting economic challenges during the pandemic which required financial state support, e.g., for closing businesses, and for people working only part-time during the pandemic. The strong increase in federal government debt was promoted by a comprehensive economic stimulus package, amounting to €130 billion euros in June 2020 and an additional €30 billion euros in November 2020 (https://www.bundesfinanzministerium.de/Web/EN/Issues/Priority-Issues/stimulus-package-for-everyone/stimulus-package-for-everyone.html, accessed on 27 September 2021). On the other hand, unemployment would have been significantly higher without such support being provided to self-employed individuals, companies, associations, and institutions in the form of direct grants, state guarantees for loans, or subsidised public loans to safeguard employment (https://ec.europa.eu/commission/presscorner/detail/en/IP_20_2180, accessed on 20 November 2020). According to the Federal Statistical Office of Germany (https://www.destatis.de/EN/Themes/Economy/Short-Term-Indicators/Labour-Market/arb210a.html, accessed on 27 September 2021), following August 2020, the unemployment rate declined slightly between September 2020 (6.2%) and December 2020 (5.9%). This relatively positive development regarding unemployment during a time when infection rates and general economic challenges appeared to be on the rise might explain why the number of unemployed people was not significantly associated with increasing psychological distress over the 12 months of COVID-19 in Germany. Previously, we found a rapid rise in the unemployment rate in Germany from March (5.1%) to August (6.4%) 2020, which was associated with poor mental health during the first wave and the onset of the second wave of the pandemic [15]. Although economic projections have generally improved since early in the recession, the nonpartisan Congressional Budget Office in the United States has projected that unemployment rates over 5.0% will persist over the next two years (https://fas.org/sgp/crs/misc/R46554.pdf, accessed on 20 August 2021). A potential contraction in economic growth following the pandemic may reinforce the inequalities of social stratification and afflict individuals or societies by means of a transition into a novel economic situation in some sectors (e.g., doorstep delivery, video conferencing and online learning, telemedicine, and onliness fitness) over the next few years [26,35]. With fears surrounding forthcoming economic challenges [5], especially in undeveloped and developing countries, people may suffer from economic recession and personal distress above and beyond infection rates [6,36].

Our study revealed that the number of active COVID-19 cases in Germany were statistically significantly associated with increased perceived distress over 12 months from 2020 to 2021. The number of active cases of COVID-19 in Germany increased significantly from a monthly average of 72,273 active cases in October 2020 up to 356,223 active cases in the second wave of COVID-19 by December 2020. In a previous six-month study from April to September 2020 when confronted with the first wave, we did not find a significant association between the number of active COVID-19 cases and perceived psychological distress [15], which might be due to the rather low COVID-19 case numbers and even a decrement in cases from 54,175 cases in April 2020 to 19,023 cases September 2020. A sustained rise in infections during the second wave may explain why our society is afflicted by the pandemic, particularly so as it hit its peak over the Christmas period [37]. Travel and contact restrictions, a cancellation of the Christmas markets, and a ban on New Year’s Eve fireworks in Germany all limited the potential for social meetings and contact, and provided a challenging situation, especially for younger people [37].

Our study highlights the importance of individual differences with regard to mental health challenges as associated with the pandemic. We found that low income and a high prevalence of loneliness were associated with increased levels of experienced psychological distress. Our findings are consistent with previous observations that individuals with lower income are particularly at risk of experiencing high levels of loneliness and subsequently poor mental health during the COVID-19 pandemic [28]. This effect is especially prominent among females and younger age groups [29]. Moreover, an ecological momentary study showed that there was a day-to-day carry over effect of negative mood and worry related to COVID-19, feeling restricted by COVID-19, and that feeling lonely increased negative mood [38].

Our current results remain tentative due to the lack of assessments pertaining to daily experiences, behaviour, and moment-to-moment fluctuations in individuals’ mental states in daily life. Our survey did not inquire about debt levels owed by individuals or households, so we were unable to detect more subtle impacts of private debt on psychological distress. We did not use an offline survey, so our study did not include anyone without internet access. In addition to internet access, the limitations of internet-based surveys include random sampling validity, lack of behavioural measures beyond reliance on self-report measures, lack of financial incentives for participation, and lack of follow up [39,40,41]. Generalizability and representativeness are limited in our sample (e.g., a high rate of female participants and no gender diverse groups), although our socio-demographic characteristics are consistent with other published surveys during COVID-19 [14]. Moreover, we did not assess and diagnose existing psychiatric conditions, such as depression, anxiety or suicide ideation, which have been included in a previous study on the association between unemployment and depression and suicide ideation in Italy [42].

Our study is clinically relevant and reveals several practical implications. The government may need to increase the level of investment in essential mental health services during or after the pandemic [43]. Psychiatrists and mental health professionals may be required to play an active role in informing policy makers about possible long-term mental health impacts and the related increasing demand for mental health service [43]. Policy makers need to envisage the influences of socioeconomic determinants of mental health, which could be of particular importance in the aftermath of COVID-19 [44]. An increased demand for mental health services is likely to persist for a long period of time, given the association between economic hardship and mental health problems [45]. A rapid investment in social protection measures (e.g., income security and bankruptcy safeguard) may protect mental health in the short term [46]. However, this can only be achieved through adequate resourcing for those measures. A key step to alleviate the mental health burden of such factors is to identify and target specific and high-priority economic sectors with the greatest immediate unemployment risks in the post-COVID-19 era [45]. This is also relevant given that economic recovery depends on a resilient workforce, something that is promoted by mental health recovery support [45].

In summary, our study highlights the long-term impact of general societal factors as well as individual socioeconomic factors on psychological distress during the pandemic. Limited financial resources and high levels of loneliness were significantly associated with increased psychological distress over the first 12 months of COVID-19 in Germany. Ongoing efforts akin to the “Campaign to End Loneliness” in the United Kingdom and “Connect2Affect” in the United States can be very powerful tools to increase awareness, community resources and support for individuals amid a crisis that may lead to social isolation and loneliness [47]. Moreover, psychological empowerment can also prevent mental health problems arising from a fear of COVID-19 [48]. Challenging times like these call for mutual support and collective resilience in the face of crisis.

## Figures and Tables

**Table 1:** Participants’ sociodemographic variables and group comparisons.

	Group	*n*	Mean Age (SEM)	*p*	Mean Education (SEM)	*p*	Mean CPDI (SEM)	*p*
Gender	Male	699	36.91 (0.53)	0.301	16.37 (0.15)	0.327	31.39 (0.72)	<0.001
	Female	2004	36.28 (0.29)		16.54 (0.09)		34.3 (0.43)	
Age, years	Elder ^1^	1328	47.52 (0.27)	<0.001	17.28 (0.12)	<0.001	29.56 (0.52)	<0.001
	Younger ^1^	1304	25.34 (0.11)		15.65 (0.09)		37.77 (0.51)	
Education, years	Higher ^2^	1214	38.69 (0.34)	<0.001	19.96 (0.08)	<0.001	29.17 (0.49)	<0.001
	Lower ^2^	1154	34.48 (0.43)		12.96 (0.04)		37.25 (0.59)	
Income, Euro	Higher ^3^	1193	42.30 (0.35)	<0.001	17.80 (0.13)	<0.001	28.74 (0.54)	<0.001
	Lower ^3^	1068	29.39 (0.35)		15.24 (0.10)		38.71 (0.55)	
ULS-8	Higher ^4^	716	32.47 (0.45)	0.018	15.43 (0.14)	0.009	52.43 (0.60)	<0.001
	Lower ^4^	840	33.94 (0.43)		15.93 (0.13)		29.84 (0.53)	

Abbreviations: *p*—statistical significance, *n*—number of participants, SEM—Standard error of the mean, CPDI—COVID-19 Peritraumatic Distress Index, ULS-8—short-form UCLA Loneliness Scale. ^1^ For age group comparison (elder vs. younger), each participant’s age was ranked higher versus lower than the median (*n* = 1328/1304: 63 subjects’ data in the median (value of 33) were removed). ^2^ For group comparison in education levels (higher vs. lower), each participant’s years of education was ranked higher versus lower than the median (*n* = 1214/1154: 233 subjects’ data in the median (value of 16) were removed). ^3^ For group comparison in income levels (higher vs. lower), each participant’s self-reported annual net income was ranked higher versus lower than the median (*n* = 1193/1068: 442 subjects’ data in the median (category of €15.000–€24.999) were removed). ^4^ For group comparison in loneliness levels (higher vs. lower), each participant’s self-reported loneliness score (ULS-8) was ranked higher versus lower than the median (*n* = 716/840: 140 subjects’ data in the median (value of 21) were removed). To test for differences between the groups, an independent sample *t*-test was used. Two-tailed *p* values were reported).

## Data Availability

The data presented in this study are available on request from the corresponding author.
